# Miscellany

**Published:** 1895-07

**Authors:** 


### Miscellany.

THE Doliber-Goodale Company appears in our columns for the first time, in this issue, advocating the special claims of Mellin's food to professional favor. Mellin's food has been long and favorably known as a valuable food for infants, children and adults.

THE Crystal Water Company, of Buffalo, announces to the profession, through our columns for the first time, in the present issue, that they manufacture a pure water for table, family and medicinal uses.

